# Biomechanical Effects of Different Auxiliary-Aligner Designs for the Extrusion of an Upper Central Incisor: A Finite Element Analysis

**DOI:** 10.1155/2019/9687127

**Published:** 2019-08-07

**Authors:** R. Savignano, R. Valentino, A. V. Razionale, A. Michelotti, S. Barone, V. D'Antò

**Affiliations:** ^1^AirNivol s.r.l., Via Giuntini 25, 56023 Navacchio, Pisa, Italy; ^2^Department of Neurosciences, Reproductive Sciences and Oral Sciences, University of Naples Federico II, Via Pansini 5, 80131 Naples, Italy; ^3^Department of Civil and Industrial Engineering, University of Pisa, Largo Lucio Lazzarino, 56122 Pisa, Italy

## Abstract

**Aim:**

To evaluate the biomechanical effects of four different auxiliary-aligner combinations for the extrusion of a maxillary central incisor and to define the most effective design through finite element analysis (FEA).

**Materials and Methods:**

A full maxillary arch (14 teeth) was modelled by combining two different imaging techniques: cone beam computed tomography and surface-structured light scan. The appliance and auxiliary element geometries were created by exploiting computer-aided design (CAD) procedures. The reconstructed digital models were imported within the finite element solver (Ansys® 17). For the extrusion movement, the authors compared the aligner without an attachment with three auxiliary-aligner designs: a rectangular palatal attachment, a rectangular buccal attachment, and an ellipsoid buccal attachment. The resulting force-moment (MF) system delivered by the aligner to the target tooth and the tooth displacement were calculated for each scenario.

**Results:**

The maximum tooth displacement along the *z-*axis (0.07 mm) was obtained with the rectangular palatal attachment, while the minimum (0.02 mm) was obtained without any attachments. With the ellipsoid attachment, the highest undesired moments *M*_*x*_ and *M*_*y*_ were found. The rectangular palatal attachment showed the highest *F*_*z*_ (2.0 N) with the lowest undesired forces (*F*_*x*_ = 0.4 N; *F*_*y*_ = −0.2 N).

**Conclusions:**

FEA demonstrated that the rectangular palatal attachment can improve the effectiveness of the appliance for the extrusion of an upper central incisor.

## 1. Introduction

The extrusion of the anterior teeth is required to treat a challenging malocclusion: the anterior open bite. This malocclusion has a multifactorial aetiology and skeletal or dental components [1–3].

When aligners are used to treat open bite malocclusion, the extrusion of the anterior teeth, the intrusion of the posterior teeth, or both orthodontic movements are required [4].

Concerning orthodontic extrusion, previous studies about clear aligner therapy reported that movements on the vertical plane compared to the other movements have larger deviations and lower predictability [5, 6]. Previous studies reported the lowest accuracy for the extrusion movement (average value 29.6%); this value was 18% for a maxillary central incisor and 25% for a mandibular central incisor [7]. The low accuracy of this movement is due to a poor grip of the aligner with the target tooth during the vertical pull. To overcome these limitations, some author proposed using attachments or elastic from a button bonded onto the tooth's buccal surface [7, 8].

Even though both fixed appliances and clear aligners can move the teeth to clinically acceptable positions, there is no literature on the force magnitude that is being created by aligner therapy [9].

Previous studies showed methodological deficiencies related to the sources of bias, such as the study design, sample size, and the lack of a control group. Most of these were retrospective studies, based on superimpositions of the initial and final cast models [6, 7, 10].

Kravitz and coauthors calculated tooth movement accuracy by the superimposition of the virtual model of the predicted tooth position over the virtual model of the achieved tooth position obtained from posttreatment impressions. To overlap the two digital models, the authors refer to the untreated teeth. By using this methodology, the teeth can be superimposed within an accuracy of 0.2 and 1.0 mm [7, 11, 12].

These methods used in previous studies to evaluate the predictability of tooth movement by aligners were not accurate for predicting the achievable movement in a specific patient nor do they take into account the root surface of the target teeth.

In the orthodontic field, a numerical simulation could provide quantitative and detailed data on the biomechanical response occurring during treatments [13]. In particular, finite element analysis (FEA) represents an effective tool to analyse orthodontic features and optimize their design. However, very few attempts have been made to study tooth-aligner interactions by finite element models (FEMs) [14–16].

The aim of this study was to design a FEM to evaluate the biomechanical effects of four different attachment-aligner configurations simulating the extrusion movement of an upper central incisor.

## 2. Materials and Methods

The computer aided engineering (CAE) workflow, used to analyse the biomechanics of the different aligner configurations, was designed according to the following steps:

1. Digital reconstruction of patient's anatomical tissues
2. Design of aligner and auxiliary elements
3. Definition of the finite element model
4. Mechanical proprieties assignment
5. Definition of boundary conditions
6. Finite element analysis

**Figure 1:**
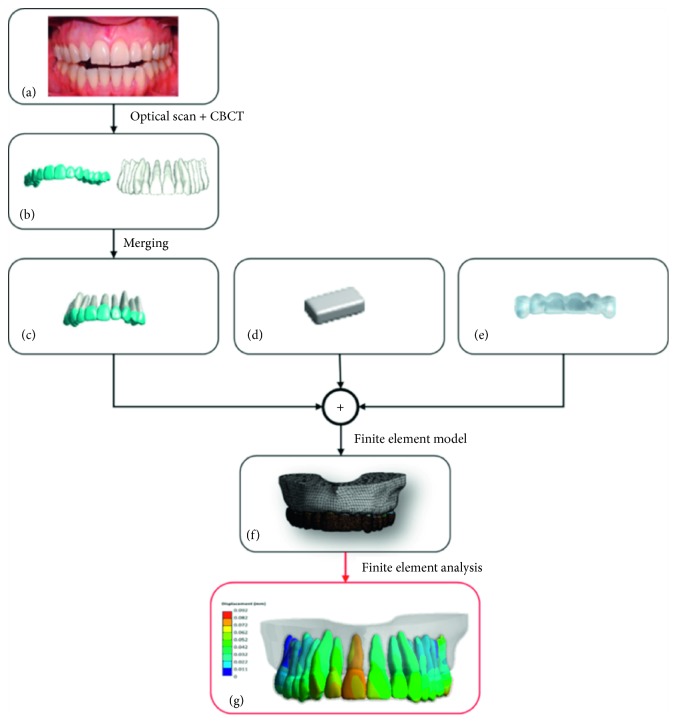
The patient's anatomical structure (a) are digitalized through optical scanning and CBCT (b). Afterwards, the two datasets are merged to create highly accurate reconstructed teeth (c). Meanwhile, attachment (d) and aligner (e) are designed through CAD operations. All the digital volumes are meshed into FEM (f), and the different configurations are simulated and analysed (g).

Figure 1 summarizes the described workflow.

### 2.1. Digital Reconstruction of Patient's Anatomical Tissues

The digital anatomical model representing all patients' tissues was obtained by using a computational and engineering framework described by Barone et al. [17].

An upper full-arch digital model was reconstructed by combining two different imaging techniques: cone beam computed tomography (CBCT) and surface-structured light scanning.

All visible dental and oral soft tissues were digitalized by using an optical scanner based on a coded structured light approach. At first, a clinician acquired a patient's upper arch impression using polyvinyl siloxane (PVS), which then resulted in a plaster model. By using the optical scanner DentalScan (Scan system srl, Navacchio, Italy), with a 10 *μ*m accuracy, a digital model composed of tooth crowns and oral soft tissues was created.

Individual crown geometries and the gingiva were obtained by a segmentation of the overall surface representing teeth shapes and oral soft tissues. The teeth and soft tissue segmentations were performed by using noncommercial software developed by AirNivol® (Navacchio, Italy). A semiautomated procedure was used, which exploits the curvature of the digital mouth model [18].

To reconstruct tooth roots and alveolar bone tissues, the authors used a sequence of Digital Imaging and Communications in Medicine (DICOM) images obtained from the CBCT technique. The patient's dental model was then obtained by merging the images obtained from the CBCT sensor and those obtained from the optical scanning. The final digital model was made of tooth crown images reconstructed by the optical scans and tooth root anatomies obtained by CBCT imaging.

This method allowed to obtain a high resolution of the crowns, which is crucial to define a precise finite element model (FEM).

CBCT data were also processed to reconstruct the alveolar bone using Amira® (Visage Imaging Inc., Carlsbad, CA).

For each slice, the regions outlined by the detected tooth contours were subtracted from the area outlined by the extracted bone contour. Teeth shapes were excluded from the alveolar bone model and replaced by the separated segmented teeth models. Therefore, each tooth could be independently manipulated within the orthodontic model, thus providing an effective tool for orthodontic simulations and treatment planning processes [19].

Periodontal dental ligament (PDL) tissues cannot be easily visualized and reconstructed by using CBCT because the slice thickness is similar to or even greater than the ligament space, which was about 0.2 mm [20].

For this reason, the PDL geometry was modelled by detecting the interface area between bone and tooth models. The PDL was simplified, neglecting its variable thickness, and modelled as a 0.2 mm uniform thick layer [16, 21]. A shell of 0.2 mm thickness was added to the external surface of each tooth; the shell volume was then subtracted from the alveolar bone to define the PDL volume [22].

### 2.2. Design of Aligner and Auxiliary Elements

For the simulation of the extrusion movement of an upper central incisor, in this study, the authors compared four different attachment-aligner configurations. A standard aligner without auxiliaries was compared to aligners designed with three different attachment shapes and positions:

- Rectangular palatal attachment (2.0 mm height × 4.0 mm width × 1.5 mm depth)
- Rectangular buccal attachment (2.0 mm height × 4.0 mm width × 1.5 mm depth)
- Ellipsoid buccal attachment (2.5 mm height × 4.0 mm width × 1.5 mm depth)

The attachments were placed with the central point of their base surface located 1 mm above the clinical crown center in the *z*-axis direction.

CAD procedures were used to create the aligner and attachment shapes as described by Barone et al. [14]. The aligner was supposed to have a uniform 0.7 mm thickness as done in previous studies [15, 16], which originates from the mean thickness of the thermoplastic material disk (0.75 mm thick) before the thermoforming process [23].

### 2.3. Definition of the Finite Element Model

The reconstructed digital models were imported within the finite element modeler, Ansys® 17 (Ansys, Inc., Canonsburg, PA). All bodies were meshed by using solid elements (quadratic 10 node tetrahedral elements). For this study, a full maxillary arch composed of 14 teeth was modelled. The mesh was constituted by approximately 900000 nodes and 480000 elements. In particular, the aligner was meshed with approximately 240000 nodes and 140000 elements, with slight differences due to the attachment shape. The PDL of the central incisor was meshed with 40000 nodes and 22000 elements. The mean size used for these models was 0.5 mm. The size of the elements has been chosen in accordance with previous studies [14, 15, 19], considering that the main purpose of this work is to compare effects of different auxiliaries on tooth movement.

The geometrical discrepancies between teeth and appliance, which result in an initial mismatch between the bodies, are the main responsible for the simulation's results. Therefore, the meshing process must be precise to preserve the ideal expected tooth movement within a certain tolerance range.

### 2.4. Mechanical Proprieties Assignment

The mechanical behavior of the alveolar bone, teeth, attachments, and aligner was described by using a linear elastic model as defined by Barone et al. [14].

Moreover, the teeth and bone were supposed to be made from a homogeneous material, without discerning in the enamel, pulp, and dentin for the teeth and the cortical and cancellous for the bone.

This assumption does not seem to affect the simulation results as reported in previous studies [20, 24, 25] because of the higher stiffness of the tooth and bone compared with PDL tissues.

It is difficult to analyse in vivo the ligament's mechanical behavior because of the small size of this structure (thickness = 0.2 mm). Therefore, most of the scientific literature has investigated the mechanical properties of the PDL through experimental analyses, and several biomechanical models were developed to describe PDL properties: linear elastic, bilinear elastic, viscoelastic, hyperelastic, and multiphase [26]. However, the complex nonlinear response of the PDL does not need to be addressed while performing an analysis of the first phase of the orthodontic reaction as in the present study [13]. It is difficult to analyse in vivo the ligament's mechanical behavior because of the small size of this structure (thickness = 0.2 mm). Therefore, most of the scientific literature has investigated the mechanical properties of the PDL through experimental analyses, and several biomechanical models were developed to describe PDL properties: linear elastic, bilinear elastic, viscoelastic, hyperelastic, and multiphase [26]. However, the complex nonlinear response of the PDL does not need to be addressed while performing an analysis of the first phase of the orthodontic reaction as in the present study [13].

The removable appliances were modelled as made of a polyethylene terephthalate glycol-modified (PETG) thermoplastic disc with linear elastic mechanical response [15, 16].

The auxiliary attachments were supposed to be made of the same tooth material.

Table 1 summarizes the material properties assigned to each body.

### 2.5. Definition of Boundary Conditions

Contact interface between the teeth and aligner, which represents the most important contact surface since it is responsible for the loading condition, was set as frictionless. This is a reasonable choice due to the existent dissimilarity between the appliance's thermoplastic material and the dental biological tissue, taking into account the presence of saliva.

Moreover, previous studies demonstrated that friction does not affect the results significantly [27].

Teeth and respective PDLs were joined by a bonded contact; bonded contacts were considered also between the bone and PDL. A bonded contact corresponds to a perfect adhesion between contact surfaces with corresponding nodes that cannot separate from each other. Moreover, the absence of a mutual sliding or separation can be assumed. The bone extremities were fixed in all directions.

### 2.6. Finite Element Analysis

**Figure 2:**
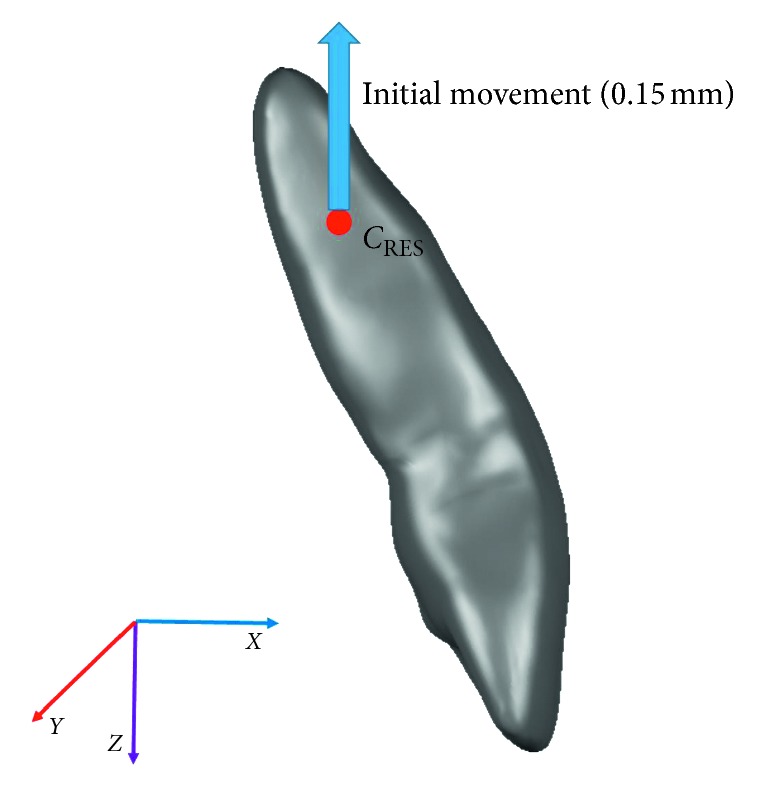
*C*_RES_ and translation imposed on the target tooth to create the initial penetration between the tooth and aligner.

The initial mismatch between the target tooth and the appliance was generated as described by Barone et al. [14], translating the target tooth, the attachment, and the related PDL and bone by 0.15 mm in the opposite direction, compared with the expected movement, as shown in Figure 2.

The nonlinear problem was solved by using the Newton–Raphson residuals method based on the force and moment convergence values.

During the Newton–Raphson iterations, the contact penetration was checked with respect to a maximum allowable penetration tolerance value, which was defined as 0.01 mm. The standard aligner led to the minimum initial penetration of 0.15 mm; therefore, a tolerance value of 0.01 mm was lower than 10% of the initial geometrical mismatch. This value was determined by considering that higher values significantly affect the results, while lower values increase convergence time without entailing significant changes in the results.

For each simulation, the resulting force system delivered by the aligner to the target tooth and the tooth displacement and rotation were calculated. The force system was calculated at the tooth's center of resistance (*C*_RES_), which was calculated according to the method described by Viecilli et al. [28]. Computational time resulted in about 6 hours for each simulation, using a workstation based on Intel Xeon CPU E3-1245 v3@3.40 GHz and 16 GB RAM.

## 3. Results and Discussion

The FEA results were analysed for each configuration by comparing forces and moments delivered to the tooth and measured at its *C*_RES_ (Table 2) and the amount and direction of orthodontic movement (Table 3).

Table 2 shows how the ellipsoid buccal attachment generated the maximum tooth displacement (0.092 mm), but in this aligner configuration, we have also found the highest undesired moments represented by mesiodistal and buccopalatal tipping (*M*_*x*_ = 2.9 N·mm; *M*_*y*_ = −1.9 N·mm). The rectangular palatal attachment showed the highest force along the extrusion axis (2 N) with lower undesired loads.

**Figure 3:**
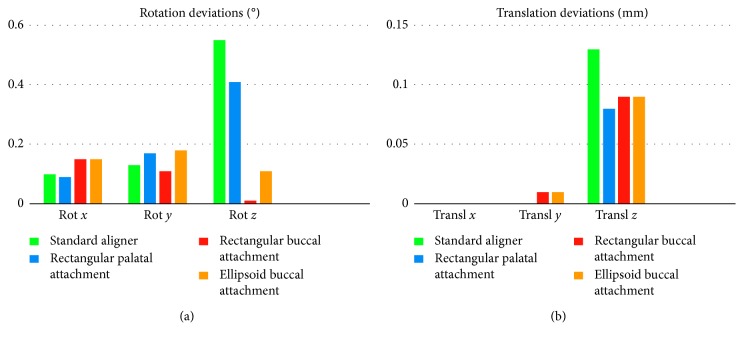
Graphical representation of rotation (a) and translation (b) deviations (absolute values) for each scenario, compared with the expected tooth movement.

Table 3 and Figure 3 show that the maximum tooth displacement along the *z*-axis was obtained with the rectangular palatal attachment, which showed 0.07 mm of translation compared to 0.06 mm obtained with the ellipsoid and rectangular buccal attachments. The lowest tooth displacement was obtained with the standard aligner configuration without attachments. The standard aligner led to the lowest desired translation on the *z*-axis, while it led to the highest undesired movement, with a rotation of −0.55° around the *z*-axis.

**Figure 4:**
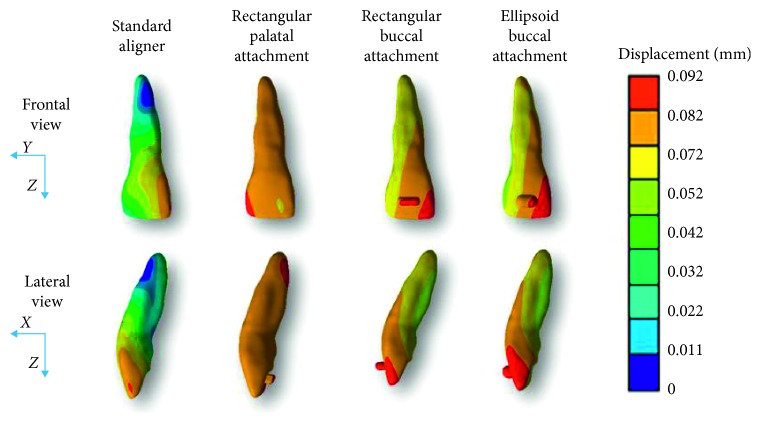
Colormap of tooth displacement for each scenario.

Figure 4 shows the total displacement for each configuration.

The analysis of the FEA results provided interesting information that could improve the design phase of orthodontic aligners. The resulting parameters of the force system helped to compare the advantages and disadvantages for each configuration. The results analysis allowed for a numeric-based decision to design the aligners. Therefore, the clinical choice for the rectangular palatal attachment would be justified by the numerical results obtained by FEA.

Few previous studies analysed tooth movements achieved by aligners by using FEM. These works calculated the force system delivered by the thermoplastic appliance to the target tooth, and they compared different aligner configurations to identify the most efficient one through a FEA [16, 29, 30]. These studies referred to different tooth movements, like canine distalization or the mesial movement of an upper molar [29, 30]. FEA results demonstrated that the different design configurations have a strong influence on the loads delivered to the target tooth. No studies analysed the extrusion movement of an upper central incisor by using the FEA before.

The analysis of the FEM results could be performed by considering three resulting outcomes:

- Force system delivered to the tooth and measured at its *C*_RES_
- Tooth translation and rotation for each spatial axis
- Colormap of tooth displacement

Table 2 shows that the highest maximum tooth displacement (0.092 mm) was obtained with the ellipsoid buccal attachment, while the lowest (0.079 mm) was obtained with the standard aligner. The maximum tooth displacement does not provide exhaustive information for analyzing the effective tooth movement because it lacks information about the movement direction. It can be considered an indicator of the amount of force and moment delivered to the tooth by the aligners, but not of the quality of the delivered loads.

For this reason, the analysis gets more consistent when referring to the force systems measured at the *C*_RES_. It can be noticed that the rectangular palatal attachment led to the highest force along the extrusion axis (2.0 N) with lower undesired moments. The other configurations brought load values lower than those with the rectangular palatal attachment and to higher undesired moments. Table 2 shows that the force delivered to the tooth along the *z*-axis (*F*_*z*_) increased by 5 times, from 0.4 N to 2.0 N, after adding the rectangular palatal to the standard aligner. However, the aligner with the ellipsoid and rectangular buccal attachments brought a lower *F*_*z*_ (1.3 N) and higher undesired moments (*M*_*x*_ and *M*_*y*_) and forces (*F*_*x*_ and *F*_*y*_).

The amount of tooth translation and rotation in each direction, shown in Table 3 and in Figure 3, confirms the previous analysis. The palatal rectangular attachments led to the highest translation along the *z*-axis (0.07 mm), while it is clear that the standard aligner does not satisfy clinical expectations, leading to a minimum translation along the *z*-axis of only 0.02 mm.

The rectangular palatal attachment delivered the highest *F*_*z*_ (2 N), which is approximately 50% more than *F*_*z*_ measured for the other attachment configurations. However, the amount of translation along the *z*-axis increased only by 0.01 mm, from 0.06 mm to 0.07 mm. This result suggests that there are other variables that should be considered in the analysis.

The attachment location seems to affect its effectiveness more than its shape. The rectangular and ellipsoid buccal attachments led to very similar results; however, placing the rectangular attachment on the lingual surface improved the outcome significantly.

This result can be related to the different angles between the attachment active surface and the tooth.

Further studies should investigate also the effect of the attachment positioning criteria on the tooth movement, focusing on the amount of active surface of the attachment.

FEA allows also for a graphical analysis, as shown in Figure 4.

The expected extrusion should be represented by a monocromatic colormap in Figure 4, meaning a pure translation of the tooth along the *z*-axis.

Therefore, the best colormap in Figure 3 is represented by the most uniform colour distribution. The rectangular vestibular attachment and the ellipsoidal vestibular attachment configurations are characterized by an evident colour modification from green to red on the *x* and *y* axes. Therefore, the tooth movement is largely represented by undesired movement. This consideration could be clearer by noticing that the palatal rectangular attachment colormap is mostly uniform.

The standard aligner is clearly the worst configuration also when considering the map of displacement. The colormap shows a large blue circle, which approximately represents the tooth's center of rotation (*C*_ROT_). Therefore, instead of a pure translation along the *z-*axis, this configuration generated mainly a rotation around *C*_ROT_.

The results show that the rectangular and the ellipsoidal buccal attachments provided similar results on the force system delivered to the tooth.

Comparing the results obtained with the buccal and palatal rectangular attachment, it is noticeable that *M*_*y*_ decreased to 0.6 N·mm compared with −1.7 N·mm and −1.9 N·mm obtained with the buccal rectangular and buccal ellipsoidal attachment, respectively. This effect can be explained by analyzing the distance between the attachment and *C*_RES_ on the *x*-axis in the 3 configurations. It is known that higher distances on the *x*-axis and *z*-axis between the force application and the *C*_RES_ generate higher *M*_*y*_.

The extrusion movement requires a force system without any moment and only one force (*F*_*z*_). Therefore, the most effective configuration should provide the aligner with the maximum contact surface on the *xy* plane to deliver *F*_*z*_. Meanwhile, the contact surface should be located as close as possible to the *C*_RES_ on each axis to avoid undesired moments.

According to our research, the design of an aligner treatment should be split into two parts: the definition of the expected movement and the choice of the best auxiliary element for each specific movement.

The aim was to demonstrate how an auxiliary element's features affect the interaction between the aligner and target tooth. Results demonstrated that attachments are crucial for improving the effectiveness of the extrusion movement. In particular, the rectangular palatal attachment can improve the effectiveness of the appliance better than the rectangular buccal attachment and the ellipsoidal buccal attachment.

Further studies should be carried on, accounting for less idealized conditions. In particular, it could be useful to use a real thermoformed aligner with nonuniform thickness, and the results should be compared to those obtained with a uniform aligner to evaluate the effect of this simplification.

Moreover, the present study analysed the first effect of the orthodontic appliance, thus assuming linear elastic mechanical behavior for all bodies.

A more complete FEA could be carried on by using nonlinear mechanical response for PDL and aligners.

CAE proved to be useful for analyzing aligner behavior and providing information to enhance their design. The paper showed how it could improve the knowledge of tooth-appliance interaction in orthodontics.

## 4. Conclusions

Considering the results obtained through the FEA, we can conclude the following:

- The extrusion of an upper central incisor cannot be achieved without any attachment.
- The shape and position of the attachments affect the expected orthodontic movement. In this case, the rectangular palatal attachment proved to be the best configuration to improve the effectiveness of the appliance for the extrusion movement of an upper central incisor.
- The attachment position, which influences the area of its active surface for the specific movement, showed a stronger influence on the outcome compared to its shape.
- The analysis of the force system delivered by the aligner to the tooth should not only focus on the desired loads but also the effect of undesired loads should be properly taken into account, as it is a determinant when selecting the proper appliance configuration.
- The developed model can well simulate the initial phase of an orthodontic treatment and can be used, during the treatment design process, for the optimization of aligner features in order to obtain a more predictable orthodontic treatment.
- Further studies should analyse the effect of nonlinear mechanical behavior of PDL and aligner and their variable thickness. Moreover, it would be useful to investigate the effect of multiple attachment positioning criteria.

## Figures and Tables

**Table 1:** Mechanical properties assigned to each body.

	Young's modulus (MPa)	Poisson's ratio
Tooth	20000	0.3
Bone	13800	0.3
Aligner	2050	0.3
Attachment	20000	0.3
PDL	0.059	0.49

**Table 2:** Maximum displacement and loads delivered to the tooth by the different aligners.

	Standard aligner	Rectangular palatal attachment	Rectangular buccal attachment	Ellipsoid buccal attachment
Maximum tooth displacement (mm)	0.079	0.088	0.086	0.092
*F*_*x*_ (N)	0.0	0.4	0.7	0.8
*F*_*y*_ (N)	0.0	−0.2	0.4	0.3
*F*_*z*_ (N)	0.4	2.0	1.3	1.3
*M*_*x*_ (N mm)	1.5	−1.7	2.8	2.9
*M*_*y*_ (N mm)	1.8	0.6	−1.7	−1.9
*M*_*z*_ (N mm)	−2.8	−1.9	1.0	0.7

“*F*” represents the force in each direction, and “*M*” denotes the moment along each direction.

**Table 3:** Translation and rotation movements of the target tooth in the four different configurations.

	Expected movement	Standard aligner	Rectangular palatal attachment	Rectangular buccal attachment	Ellipsoid buccal attachment
Rotation *x* (°)	0	0.1	−0.09	0.15	0.15
Rotation *y* (°)	0	−0.13	−0.17	−0.11	−0.18
Rotation *z* (°)	0	−0.55	−0.41	−0.01	−0.11
Translation *x* (mm)	0	0	0	0	0
Translation *y* (mm)	0	0	0.01	0.01	0.01
Translation *z* (mm)	0.15	0.02	0.07	0.06	0.06

## Data Availability

No data were used to support this study.
